# Transcriptome Profile of the Chicken Thrombocyte: New Implications as an Advanced Immune Effector Cell

**DOI:** 10.1371/journal.pone.0163890

**Published:** 2016-10-06

**Authors:** Farzana Ferdous, Christopher Saski, William Bridges, Matthew Burns, Heather Dunn, Kathryn Elliott, Thomas R. Scott

**Affiliations:** 1 Department of Animal and Veterinary Sciences, Clemson University, Clemson, South Carolina, United States of America; 2 Clemson University Genomics Institute, Clemson University, Clemson, South Carolina, United States of America; 3 Department of Mathematical Sciences, Clemson University, Clemson, South Carolina, United States of America; 4 Clemson Cooperative Extension, Clemson University, Clemson, South Carolina, United States of America; Institute of Oceanology, Chinese Academy of Sciences, CHINA

## Abstract

Thrombocytes are nucleated platelets involved in immune functions such as pathogen recognition and release of pro-inflammatory bioactive compounds when exposed to bacterial and viral molecules. However, the complete role of these cells in innate and adaptive immune responses is not understood, and little is known about their biology at the molecular-genetic level. Highly sensitive RNA-sequencing technologies were used to analyze the complete transcriptome of thrombocytes for the first time with analytical resolution focused on cell-based components of the immune system/response. Amongst all the genes listed in the current chicken genome assembly, 10,041 gene transcripts were found in the chicken thrombocyte. After 1-hour *in vitro* stimulation with lipopolysaccharide (LPS, *Salmonella minnesota*), 490 genes were upregulated and 359 genes were downregulated, respectively, with at least a 1-fold change relative to unexposed thrombocytes. Additionally, by constructing a *de novo* assembly, we were able to identify a total of 3,030 novel genes in the thrombocyte transcriptome. The information generated here is useful in development of novel solutions to lower the economic burden and zoonotic threat that accompanies infectious diseases for birds and fish. In addition, the resources created here have translational utility as a model system to find orthologous genes and genes related to its enucleated counterpart, the platelet.

## Background

Thrombocytes are smallest of the blood cells and are found in lower vertebrates such as reptiles, amphibians, fish, and birds [1]. These cells are nucleated and considered the first cells to evolve that specialize in hemostasis [1–3]. Nonnucleated thrombocytes or platelets are only found in mammals [1]; and thrombocytes are functionally comparable and primarily involved in hemostatic functions/wound healing. In recent years, these cells have been shown to have roles in inflammation, anti-microbial host defense, and overall immune response [4–8].

The role of thrombocytes in immunity was shown with evidence of phagocytic ability, followed by a role in the inflammatory response. Thrombocytes have been shown to express, produce or release a variety of mediators of inflammation, antimicrobial activity and other immune modulating activity [6]. The discovery of Pathogen Recognition Receptors (PRRs) such as Toll-like Receptors (TLRs) on these cells has led to a new understanding of the thrombocyte role in immune responses [4,9–14]. Thrombocytes respond to lipopolysaccharide (LPS) [12,15], and this stimulation takes place through TLR4, mitogen-activated (protein (MAP) kinase (ERK, MEK1 and p38 MAPK) and nuclear factor-κ light-chain-enhancer of activated B cells (NF-κB) pathways [12].

Thrombocytes have been suggested to be the hemostatic homologue of the mammalian platelet due to combined morphologic, immunologic and functional evidence; and conservation of major hemostatic pathways involved in platelet function and blood coagulation [16,17]. Due to the importance of platelets in human medicine, a substantial amount of research has been conducted to study the role of these cells in physiological function [18–20], and the capability to be involved in the immune response [4,5,21–23] is better understood compared to the thrombocyte. However, the full capability of the platelet to influence overall physiology would be better revealed through experimentation with the thrombocyte. Thrombocytes could serve as a nucleated model to provide new insights into platelet hemostasis, thrombosis and even bleeding disorders.

Our lab has studied *in vitro* stimulation of chicken thrombocytes with bacterial and viral Toll-like receptor ligands for several years [12,24] to establish the proper role of this cell in immunity. Here, we have used RNAseq technology to characterize the global transcriptome of the chicken thrombocyte and its *in vitro* response to stimulation with Salmonella-derived endotoxin (LPS) in order to expand our knowledge about these cells. The long-term aim of our research is to generate an essential genomic resource that will have translational utility in the medical world as a model system to find orthologous genes and genes related to platelet disorders. In addition, such resources will be useful in development of novel solutions to lower the economic burden and zoonotic threat that accompany infectious diseases for birds and fish. Such solutions include the identification of biomarkers for elite disease resistance genes expressed by thrombocytes. Determination of biomarkers by examining the gene expression profile of thrombocytes, stimulated with pathogenic agents, may be used as an early detection of zoonotic/infectious agents affecting young or even breeding-age poultry.

## Materials and Methods

### Chickens

Three female Single Comb White Leghorn (SCWL) chickens (16-weeks old) were randomly selected for blood collection in this study. The chickens were housed at the Clemson University Morgan Poultry Center, Clemson, SC, which is an Institutional Animal Care and Use Committee (IACUC) approved animal facility operating under standard management practices adhering to the Association for Assessment and Accreditation of Laboratory Animal Care International criteria.

### Thrombocyte Isolation and *In Vitro* Stimulation

Syringes fitted with needles were used to collect 3 mL of whole blood from the wing vein of each chicken into 0.1 mL of 10% ethylenediaminetetraacetic acid (EDTA) solution. The collected blood samples were stored on ice until brought back to the laboratory. Each blood sample was diluted (1:1) with calcium and magnesium free Hank’s balanced salt solution (HBSS) (Cambrex Bio Sciences Walkersville Inc., Walkersville, MD). Diluted blood samples were then layered on a lymphocyte separation medium (Density 1.077–1.080 g/mL, Mediatech. Inc., Herdon, VA) and centrifuged at 1700 x *g* for 30 min at 23°C to collect the thrombocyte-rich band as previously described by Scott and Owens [12]. The isolated thrombocyte enriched cell suspension routinely is 99% positive for the thrombocyte specific marker CD41/61 [14,25]. Trypan blue solution (0.4% w/v in normal saline) was used for quantification of viable cell numbers on a SPolite^®^ Hemacytometer (Baxter Healthcare, McGaw Park, IL) with the aid of an upright light microscope. The isolated thrombocytes from each chicken were incubated with 1 μg/mL of ultra pure LPS from *Salmonella minnesota* (InvivoGen, San Diego, CA). The control samples were incubated with only HBSS and no LPS. The cell suspensions with and without LPS were incubated in sterile 1.5 mL microcentrifuge tubes (1 x 10^7^ cells per tube) on a rocking platform (VWR, Suwanee, GA) at 41°C for 60 min. The concentration of LPS used and stimulation length was chosen based on previous experiments performed in our laboratory.

### RNA Isolation, Quantification, and Quality Assessment

For RNA isolation after thrombocyte stimulation, cells were centrifuged at 5000 x *g* for 2 min to pellet. The pellets were stored in 100 μL of RNAlater™ (Qiagen Inc., Valencia, CA), an RNA stabilizing solution. After 24 hr at 4°C in RNAlater™, the cells were centrifuged again to remove the supernatant and stored at -20°C until thawed for RNA isolation. The RNeasy^®^ Kit (Qiagen Inc., Valencia, CA) was used according to the manufacturer’s protocol to isolate the total RNA from these samples. The RNA samples were treated with an on-column DNase (Qiagen Inc., Valencia, CA) to remove any possible contamination from chicken genomic DNA. Isolated RNA samples were quantified and integrity validated on a Nano Drop 1000 Spectrophotometer (Thermo Scientific, Waltham, MA) and Bioanalyzer 2100 (Agilent Technologies).

### Illumina Library Construction, RNA Sequencing, and Analysis

Each thrombocyte sample was normalized to a standard input concentration (1 μg of Total RNA) and an Illumina compatible sequencing library was prepared robotically on a Microlab STAR (Hamilton) with the TruSeq stranded total RNA library prep kit (Illumina) following the manufacturer’s recommended procedures (Illumina). The resulting sequencing libraries were assessed for size on a 2100 Bioanalyzer (Agilent) and sequence data collected on 1 lane of an Illumina HiSeq2500 with a 2x125 bp PE read on high-output mode. Raw sequence reads were assessed for run quality with the FastQC analysis package [26], and then preprocessed to remove adapter and low quality bases with the Trimmomatic software package [27]. Processed reads were mapped to the Gallus_gallus-4.0 reference assembly (GenBank Assembly ID GCA_000002315.2) [28] with the BWA [29]. Each replicate transcriptome was plotted together as a multidimensional scaling plot to observe global sample variation (S1 Fig). The control replicate Number 2, and the LPS stimulated replicate Number 2 were removed due to high variability in the first dimension. Further analysis were performed with an n = 2 for both unstimulated and LPS stimulated conditions. Read abundance counts per exon were determined with the Subread [30] and differential gene expression determined with EDGER [31]. Gene Ontology enrichment and analysis was performed with the Panther suite of analytical tools [32]. We utilized the Panther derived gene ontology (GO-slim terms) used for broad classification of molecular function, biological processes, and cellular components[32,33]. *De novo* transcriptome assembly of the thrombocyte was performed with the Trinity [34].

## Results

### Thrombocyte Transcriptome

**Fig 1:**
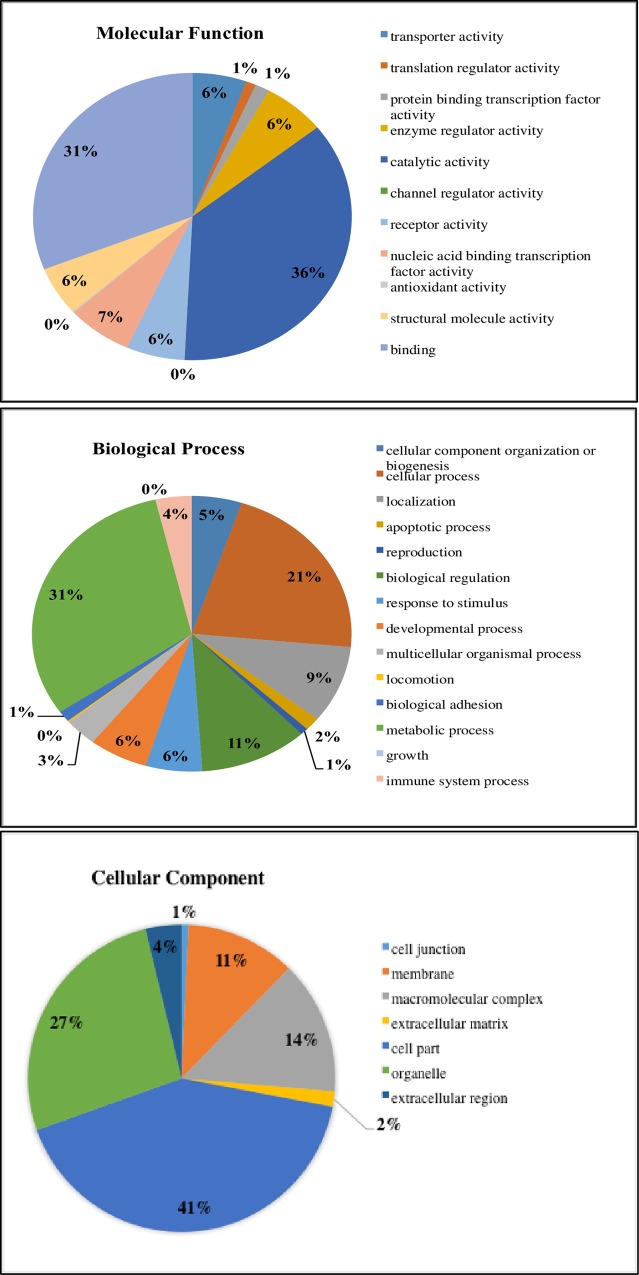
Distribution of the chicken thrombocyte transcripts in unstimulated cells categorized as cellular processes according to the Gene Ontology (GO)-slim categories of molecular function (MF), biological process (BP), and cellular component (CC).

A total of 10,041 transcripts were detected in unstimulated control chicken thrombocytes compared to the 17,108 total annotated gene sequences identified in the reference chicken annotation. In order to decipher the functional aspects of the thrombocyte genes, we organized the transcripts based on their Gene Ontology (GO) functional categories. The results indicated that these cells have a role in a broad range of different biological activities and functions (Fig 1). Within the molecular function (MF) category, the most abundant terms observed include catalytic activity, binding, and nucleic acid binding transcription factor activity, representing close to 75% of all the MF terms. Within the biological processes (BP) category, the most abundant terms detected were metabolic processes, cellular processes, biological regulation, localization, response to stimulus, and immune system processes. Within the cellular component (CC) category, 41% of the terms were related to cell part, 27% to organelle, 14% to macromolecular complex and 11% to membranes.

### Pathways and Genes Involved with Immune Function

**Fig 2:**
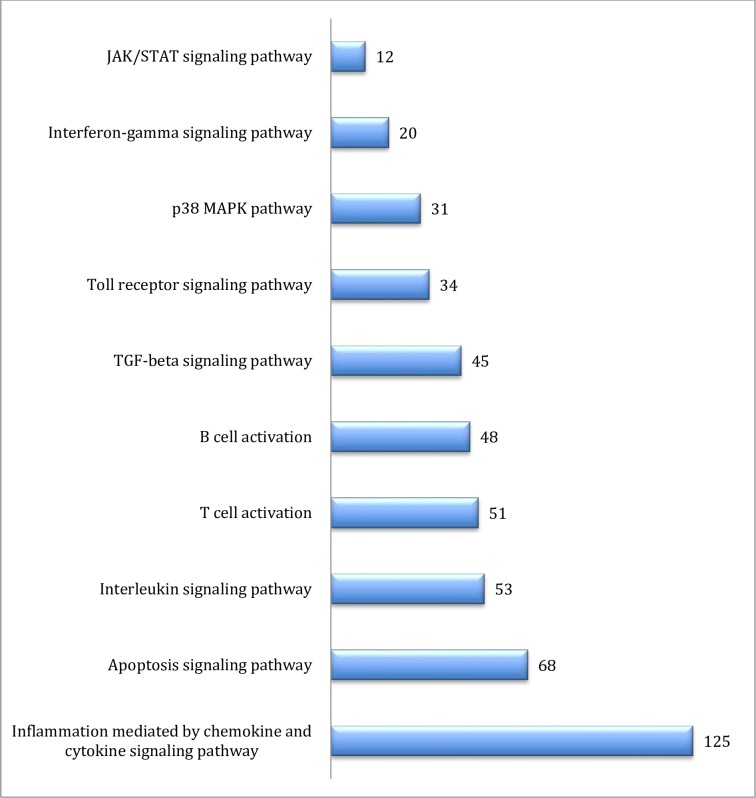
Representative number of genes in the top 10 immune-related GO-slim biochemical pathways. The numbers beside the blue bars represent the number of genes in that category. The complete list of GO-slim biochemical pathways detected in unstimulated thrombocytes are listed in S1 Table.

In order to give a broad classification of gene product function, we categorized the gene ontology content as respective biochemical pathways according to selected GO-slim terms; the cellular processes, pathways, and the number of genes associated with each of the assigned terms are listed in S1 Table. In order to further expand the knowledge regarding the role of thrombocytes in the immune system, the results presented here are directed toward the identification of biochemical pathways and genes that are involved in immune function. Upon examination of the GO-slim biochemical pathways, we identified ten pathways associated with immune signaling. We identified a total of 453 genes with roles in inflammation mediated chemokine and cytokine signaling (125), apoptosis signaling (68), interleukin signaling (53), T-cell activation (51), B-cell activation (48), transforming growth factor beta (TGF-β) signaling (45), TLR signaling (34), p38 mitogen-activated protein kinase (MAPK) signaling (31), interferon-gamma (IFN-γ) signaling (20) and Janus kinase/signal transducers and activators of transcription (JAK/STAT) signaling (12, Fig 2).

In addition to TLR signaling genes, we detected gene transcripts for nucleotide binding oligomerization domain (NOD)-like receptors (NLRs) such as NLR Family Member X1 (NLRX1), NLR Family CARD Domain Containing 3 and 5 (NLRC3, NLRC5) in the thrombocyte transcriptome. According to our RNAseq data, thrombocytes expressed MHC class I alpha chain 2 (such as BFIV21), and MHC class II genes (such as BLB1, BLB2, B-MA2). Many genes associated with major histocompatibility complex I and II, antigen processing and presentation were found in thrombocytes (S2 Table) including transcripts for accessory molecules CD40 and CD80 that are found on antigen presenting cells (APCs) in unstimulated thrombocytes. Furthermore, a broad search of the GO terms for “immune” revealed 244 genes with this assigned annotation in both the biological process and molecular function categories (S3 Table).

### Transcriptional Response to LPS

**Fig 3:**
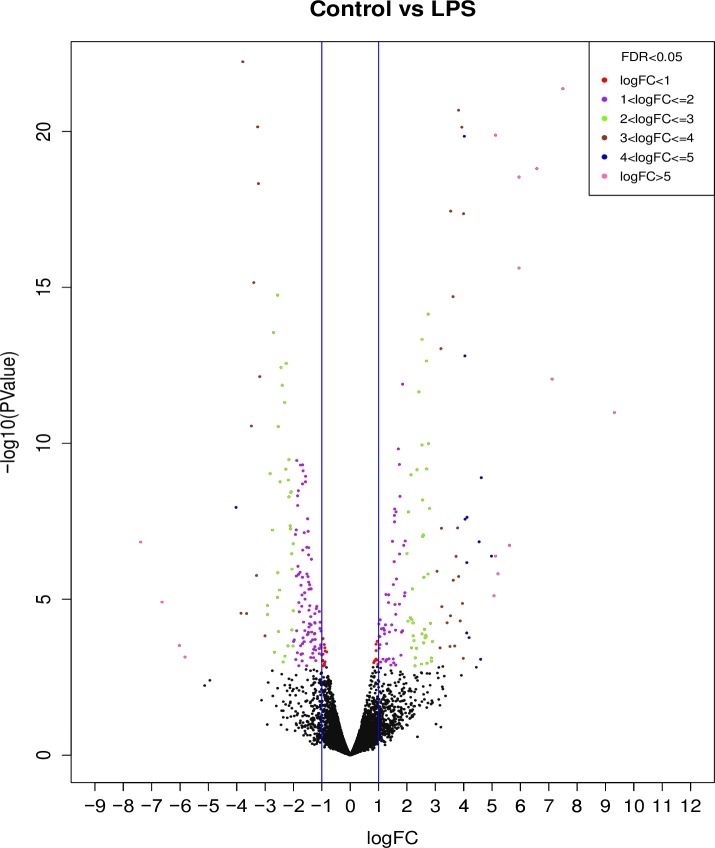
A volcano plot of genes with differential expression profiles after a 1 hr treatment with LPS. Positive values on the X-axis indicate genes with increased transcript abundance, and negative values indicate genes with decreased transcript abundance. Black dots indicate genes that are greater than the false discovery rate (fdr) of 0.05, and the vertical blue bars delineate a threshold of 1 fold change.

Upon stimulation with LPS, we detected evidence for a total of 10,148 genes being transcribed, of which, 354 are unique to this treatment relative to control thrombocytes (S4 Table). Differential expression profiling yielded upregulation of transcription for 490 genes and downregulation of 359 transcripts relative to the unstimulated control cells (Fig 3, S5 Table). The top 10 biochemical pathways up and downregulated in response to LPS stimulation are shown in Table 1. The upregulated pathways include Wnt signaling, inflammation mediated by chemokine and cytokine, cholecystokinin signaling map, angiogenesis, interleukin signaling, integrin signaling, platelet-derived growth factor signaling, cadherin signaling while the downregulated pathways include inflammation mediated by chemokine and cytokine, TGF-β signaling, angiogenesis, Huntington disease, Wnt signaling and others (complete list can be found in S6 Table).

**Table 1:** Top ten categories by biochemical pathway using up/down regulated genes in response to LPS stimulation. (The complete list can be found in S6 Table.)

Upregulated	Downregulated
Wnt signaling pathway	Inflammation mediated by chemokine and cytokine signaling pathway
Inflammation mediated by chemokine and cytokine signaling pathway	TGF-beta signaling pathway
CCKR signaling map	Angiogenesis
Angiogenesis	Huntington disease
Interleukin signaling pathway	Wnt signaling pathway
Integrin signaling pathway	Parkinson disease
PDGF signaling pathway	PDGF signaling pathway
Cadherin signaling pathway	Gonadotropin releasing hormone receptor pathway
Axon guidance mediated by semaphorins	Metabotropic glutamate receptor group III pathway
Alzheimer disease-presenilin pathway	Ionotropic glutamate receptor pathway

The PANTHER overrepresentation test shows that among all three categories (GO biological process, PANTHER protein class and GO molecular function) immune related processes or proteins are upregulated (Table 2). Under GO biological processes, neutrophil and granulocyte migration and chemotaxis have the highest fold enrichment. Chemokine and chemokine activity are the most upregulated in terms of fold enrichment in PANTHER protein class and GO molecular function, respectively.

**Table 2:** Panther Over Representation Test.

	Fold Enrichment	P value
**GO Biological Process**		
neutrophil migration	8.98	2.45E-02
neutrophil chemotaxis	8.98	2.45E-02
granulocyte chemotaxis	8.44	3.85E-02
granulocyte migration	8.44	3.85E-02
defense response	2.84	7.88E-04
immune response	2.78	2.87E-03
**PANTHER Protein Class**		
chemokine	14.33	1.65E-04
cytokine	4.62	6.08E-05
signaling molecule	2.17	1.60E-04
**GO Molecular Function**		
chemokine activity	14.5	5.06E-03
cytokine activity	3.66	2.08E-03
receptor binding	2.29	7.71E-05

The top 10 upregulated and downregulated genes are listed in Table 3. Among the upregulated transcripts, IL-6 and IL-1β have roles in inflammation; IL-8 has roles in mediating cell activation and migration, and CSF3 influences granulocyte production. Among the genes with extreme downregulation profiles, we identified Glycerol-3-phosphate dehydrogenase, Rho GTPase Activating Protein 20, Semaphorin VIB, von Willebrand Factor A Domain Containing 1, Glutamate Decarboxylase 1, RasGEF Domain Family, Member 1B, Growth Arrest-specific-2, Apolipoprotein A-I, and several other genes with diverse functional roles (S5 Table). In addition to genes shown in Table 3, there were many more genes that were up or downregulated when thrombocytes were stimulated with LPS (S5 Table).

**Table 3:** Top 10 up- and down-regulated genes in response to *in vitro* stimulation of chicken thrombocyte.

ENSGALG ID	Gene	Transcript Name	logFc^•^	Known or possible function
ENSGALG00000010915	IL6	Interleukin 6	9.63	Inflammatory response and b cell maturation
ENSGALG00000026420	CSF3	Colony Stimulating Factor 3	9.31	Cytokine that controls various aspects of granulocyte production
ENSGALG00000005616	ARSI	Arylsulfatase Family, Member	7.49	Hormone biosynthesis, cell signaling
ENSGALG00000011668	K60/ IL8L1	Interleukin-8 precursor/ interleukin 8-like 1	7.25	
ENSGALG00000005619	F3	Coagulation Factor III	7.12	Cell surface glycoprotein, affinity receptor for F7
ENSGALG00000026098	IL8	Interleukin-8	6.74	Mediates activation and migration of neutrophils
ENSGALG00000006106	TNFRSF6B	Tumor Necrosis Factor Receptor Superfamily, Member 6b	6.58	Suppression of cell death
ENSGALG00000000534	IL1B	Interleukin 1, Beta	6.54	Induction of COX2, mediator of the inflammatory response
ENSGALG00000021025	LOC419276	Bactericidal permeability-increasing protein-like	5.95	Antibacterial response
ENSGALG00000016919	IRG1	Immunoresponsive 1 Homolog	5.95	Negative regulator of TLR mediated responses
ENSGALG00000012061	GPD1	Glycerol-3-phosphate dehydrogenase	-7.38	a component of glycerophospholipids.
ENSGALG00000004827		Uncharacterized	-6.63	
ENSGALG00000017150	ARHGAP20	Rho GTPase Activating Protein 20	-6.31	related pathways are Signaling by GPCR and Signaling by Rho GTPases.
ENSGALG00000001171	SEMA68	Semaphorin VIB	-6.02	may be involved in both peripheral and central nervous system development
ENSGALG00000001527	VWA1	Von Willebrand Factor A Domain Containing 1	-5.82	appears to play a role in cartilage structure and function
ENSGALG00000024466	CC17	Uncharacterized protein	-5.13	
ENSGALG00000009589	GAD67	Glutamate Decarboxylase 1 (Brain, 67kDa)	-4.95	responsible for catalyzing the production of gamma-aminobutyric acid from L-glutamic acid
ENSGALG00000010903	RASGEF1B	RasGEF Domain Family, Member 1B	-4.18	Guanine nucleotide exchange factor
ENSGALG00000028373	GAS2L3	Growth Arrest-Specific 2 Like 3	-4.02	Cytoskeletal linker protein
ENSGALG00000007114	APOA1	Apolipoprotein A-I	-3.85	Participates in the reverse transport of cholesterol from tissues to the liver for excretion by promoting cholesterol efflux from tissues and by acting as a cofactor for the lecithin cholesterol acyltransferase

^*^(logFC indicates the log2 of the fold-change between the treated and untreated samples)

### Thrombocyte Transcriptome *de novo* Assembly

In an effort to search for novel genes expressed in the thrombocyte transcriptome that are not currently annotated in the *Gal gal* 4 reference assembly, we performed a *de novo* transcriptome assembly and removed known annotated chicken genes (See Methods). After strict assembly and filtering criteria, we identified a total of 3,030 putative new coding transcripts (S7 Table). A blastx alignment to the SwissProt database revealed that only 780 of these putative genes do not produce a hit at a 1e-05 threshold. Alignment to the Gene Ontology produced a total of 1,857 hits (only 308 unique terms) (S8 Table). Molecular function included genes with roles in RNA/DNA binding, aspartic-type endopeptidase activity, and protein binding. The biological process category contained genes with roles in transmembrane kinase signaling, cellular process, and nucleobase-containing compounds (S8 Table). Moreover, a search of the GO terms for “immune” revealed a total of 80 genes (S7 Table). Among these, we identified a B-cell antigen receptor complex associated protein, several complement decay-accelerating factors, interleukins, T-cell receptors, and numerous transcription factors and other signaling molecules (S7 Table). Of the 3,030 putative new gene sequences, a total of 83 genes displayed an increase of at least a fold change of 2, and only 53 displayed a decrease in transcriptional abundance with a fold change of at least 2 when assayed for expression changes under LPS stimulation (S9 Table). Among the putative novel genes that appear to be upregulated, we identified genes with homology to interleukins, RAS-GTPase, Filamin-C, and other growth factor like genes (S9 Table). Novel genes that appear to have a decrease in expression profiles include snRNA-like, methyltransferase-like genes, among others.

## Discussion

This is the first analysis of the complete transcriptome of the thrombocyte. Analysis of GO functional categories demonstrated that these cells have a role in a broad range of different biological activities and functions. For this paper, we focused on processes, pathways, and genes related to immune response; particularly those affected by LPS exposure.

Among all the gene transcripts detected, GO-slim biological processes showed 466 genes related to immune system processes (S1 Table). Other biological process categories such as response to stimulus, and biological regulation may also have genes indirectly related to immune response. Among all biochemical pathways shown by GO-slim analysis (Fig 2), the greatest number of genes (125) was associated with inflammation mediated by chemokine and cytokine signaling pathways. These genes are primarily associated with the metabolic process, cell communication, response to stimulus, immune response, and inflammation. Apoptosis and p38 MAPK signaling genes generally participate in the signaling cascade that controls cellular responses to cytokines and stress. The TGF-β signaling pathway is commonly involved in regulation of fundamental cell processes such as proliferation, differentiation, death, cytoskeletal organization, adhesion, and migration [35]. Cellular effects of IFN-γ include up-regulation of pathogen recognition, antigen processing and presentation, the antiviral state, inhibition of cellular proliferation and effects on apoptosis, activation of microbicidal effector functions, immunomodulation, leukocyte trafficking and integration of signaling and response with other cytokines [36]. The JAK/STAT pathway is the principal signaling mechanism for a wide array of cytokines and growth factors [37]. TLR signaling is also among the GO-slim analysis of biochemical pathways observed in the thrombocyte transcriptome. TLR signaling is activated by pathogen associated molecular pattern leads to immediate innate immune responses preventing spread of infection and in the potentiation and direction of the later responses of acquired immunity [38]. A preliminary evaluation of transcripts for TLR pathway components linked to LPS stimulation of thrombocytes via TLR4 provides a characteristic set of signals leading to gene expression of pro-inflammatory mediators (i.e., IL-1β, IL-6, IL-8).

In addition to genes related to TLR signaling, we detected transcripts for NLRs. NLRs along with TLRs and others (such as mannose receptors, C-type lectin receptors, RIG-I-like receptors) are involved in the innate pathogen pattern recognition system. Among NLRs detected in the thrombocyte, NLRX1 is known to be a regulator of mitochondrial antivirus responses [39], and NLRC3 is a cytosolic negative regulator of innate immunity [40]. NLR5 is a critical regulator of MHC class I-dependent immune responses [41]. In mice, deficiency of NLR5 expression has been associated with impaired MHC class I expression, and impaired CD8^+^ T-cell activation [42]. Human NLR5 has a role in anti-viral innate immune responses[43]. NLRs have been shown to respond to intracellular pathogens and play important roles in distinct biological processes ranging from regulation of antigen presentation, sensing metabolic changes in the cell, modulation of inflammation, embryo development, cell death, and differentiation of the adaptive immune response [44].

Based on our experience in working with thrombocytes and previous studies done in our laboratory [12,14,15,24], stimulation with 1 μg/mL of LPS for 1 hr is more than sufficient to induce these cells. The most upregulated gene transcript is IL-6 when thrombocytes are exposed to LPS for 1 hr. IL-6 is a pro-inflammatory cytokine that also induces the synthesis of acute phase response proteins, terminal differentiation of B cells to antibody producing plasma cells, differentiation of monocytes to macrophages, and growth of hematopoietic stem cells [45]. Colony stimulating factor (CSF) 3 is the next most upregulated gene. CSF3 controls the production, differentiation, and function of mature granulocytes [46]. IL-8 precursor and IL-8, which is a chemoattractant, were also among the upregulated genes. IL-8 is a proinflammatory cytokine that is involved in activation and migration of neutrophils (heterophils) during inflammation [47]. Bactericidal permeability-increasing protein-like (LOC4192760) and immunoresponsive 1 homolog (IRG1) genes were also among the top upregulated genes involved in immune response. Arylsulfatase Family (ARSI) was one of the genes in this list that is not directly involved in immune response. ARSI hydrolyzes sulfate esters from sulfated steroids, carbohydrates, proteoglycans, and glycolipids and is known to be involved in hormone biosynthesis and cell signaling [48]. Coagulation factor III, known to initiate the blood coagulation cascades, was the only gene from blood coagulation that was in the list of top upregulated genes.

Depending on the cytokines and other expressed cellular markers, thrombocytes may be able to activate and affect naïve T cells to differentiate into effector T cell types. Here, we were able to identify genes in thrombocytes that indicate involvement of thrombocytes in more than just innate immunity. Expression of MHC II genes and molecules is a unique finding for nucleated thrombocytes [13,14,49] since mammalian platelets are devoid of MHC class II molecules [4,14]. It is interesting to detect MHC II transcripts in unstimulated thrombocytes since this is a feature limited to true APCs such as dendritic cells. Generally, dendritic cells (DCs, professional APCs) express both class I and class II MHC molecules while macrophages and B cells must be activated to express class II. Expression of MHC II is important as the first signal for stimulation of the T cell. We have observed that thrombocytes also express co-stimulatory molecules such as CD40 and CD80 on control unstimulated thrombocytes. Co-stimulatory molecules on APCs generally bind to CD28 on T cells and act as a second signal to activate T cells. We also observed upregulation of some cytokines like IL-6 (Table 3) and IL-1β (S5 Table). We have previously reported that LPS stimulation leads to upregulation of gene expression for IL-6 and IL-12 in chicken thrombocytes [15] providing polarizing signals for T-cell activation. IL-6 promotes Th2 differentiation and simultaneously inhibits Th1 polarization through two independent molecular mechanisms [50]. Likewise, IL-12 can positively influence differentiation to a Th1 state when conditions favor more cytotoxicity. This ability to influence T cells is a hallmark of adaptive immunity in which thrombocytes most likely share with professional antigen presenting cells.

Although mammalian enucleated platelets are devoid of MHC II, the role of these cells in adaptive immunity is fairly well established[51–53]. Platelets have been shown to activate DCs *in vitro* and promote T-cell responses via CD40L [4]. Platelet-derived CD40L has been reported to support B-cell differentiation and immunoglobulin class switching in mice [53]. Platelet-derived CD40L also has been shown to augment CD8^+^ T-cell responses, both *in vitro* and *in vivo*, and to promote protective T-cell responses following infection with *Listeria monocytogenes* [54]. Among cytokines that generally affect T-cell activation and proliferation[55], we were able to detect transcripts for IL-15. We have also detected transcripts for the components of the IL-2 receptor (IL2RA, IL2RB and IL2RG), which may be used by a number of cytokines for stimulating T-cell proliferation including IL-15. We also detected IL-16 that can function as a chemoattractant and modulator of T-cell activation.

Since thrombocytes we isolated for this study were from chickens, in addition to discussing what we have observed in our dataset, we compared our thrombocyte RNAseq data with published datasets for other chicken immune cells. This will be valuable for understanding the overall role of this particular cell in the immune system of chickens. Upon performing an extensive literature search, we were able to find RNAseq data of chicken heterophil, macrophage and dendritic cells. The publicly available RNAseq data for bone marrow-derived dendritic cells and macrophages, and heterophils isolated from blood downloaded for comparative analysis were both unstimulated and LPS stimulated for 24 hr [56]. Although this varies in terms of length of stimulation time, this dataset was most similar to the overall format of our study.

Our objective was to uncover common markers for sentinel and antigen presenting cells to compare with the thrombocyte transcriptome (S10 Table). We generated a semi-quantitative table based on raw counts. This comparison provided a side-by-side examination of some key molecules such as TLRs, TLR associated molecules, costimulatory markers, MHC and cytokines in all of these cell types. Overall, gene transcripts for most of these markers were present in each cell type making the inclusion of thrombocytes relevant with important immune cells. TLR2-2, TLR5, and IRF6 transcripts were not observed in thrombocytes, Cytokine transcripts for IL-6, IL-8 and IL-12β were not seen in unstimulated control but were observed in LPS treated cells. The expression of gene transcripts for TLR, TLR associated molecules, cytokines, co-stimulatory makers and MHC (at least BFIV21 and BLB1) were similar among the four cell types. It is essential to note that this comparison was done with publicly available RNAseq data and some of the genes that may or may not be present here could be due to an incomplete chicken genome database or to point/time of sample collections.

The potential of these cells to be involved in adaptive immunity, or at least as a bridge between innate and adaptive immune responses, has been indicated previously by some researchers [13,14,57]. Tregaskes and his colleagues demonstrated, among other biologically active surface molecules and receptors, avian thrombocytes also express CD40L [57]. The discovery of functional CD40L is of vital importance in the potential modulatory capacity of thrombocytes in bridging innate immunity to the adaptive side of immune responsiveness. CD40/CD40L is a receptor-ligand pair with a central role in promoting interactions between lymphocytes and APCs such as DCs. Therefore, thrombocytes appear to be more than innate effector cells. In addition to CD40L, it has been shown that CD40, CD80, CD86 and MHC II (molecules that are generally associated with antigen presentation) were detected on thrombocytes using flow cytometry [14]. Chicken thrombocytes should not only have the ability to interact with APC, but also have the potential to play a role in antigen presentation.

## Conclusions

To the extent that we know, this is the first report on the entire identifiable transcriptome of the nucleated thrombocyte from chicken. Global transcriptome information on these cells is important due the comparative aspect for other species to provide acceptable and valuable biomedical models for platelet physiology studies in mammals. In addition, defining the role of these cells in immune responses will be useful for economically important agriculture species such as poultry and fish. Databases generated from studies like this will be useful to discover biomarkers for assessing overall animal health.

## Supporting Information

S1 Fig Multidimentional scaling plots showing variability between the replicates of control and LPS stimulated samples. (TIF)

S1 Table Thrombocyte transcriptome GO-slim molecular function, biological process, cellular component, protein class and biochemical pathway. (XLSX)

S2 Table Thrombocyte genes associated with MHC, antigen processing and presentation. (XLSX)

S3 Table Gene transcripts with the GO term “immune” in both the biological process and molecular function categories. (XLSX)

S4 Table Additional gene transcripts that are found in LPS stimulated thrombocytes. (XLSX)

S5 Table Genes upregulated with at least 1 fold change in LPS treated cells relative to control. (XLSX)

S6 Table Upregulated and downregulated genes categorized by Biochemical Pathway. (XLSX)

S7 Table Novel thrombocyte gene annotation. (XLSX)

S8 Table Functional classification of *de novo* genes. (XLSX)

S9 Table Differential gene expression of novel chicken thrombocyte genes. (XLSX)

S10 Table Cellular attributes of thrombocytes compared to other immune cells. (XLSX)
