# Correction: Transcriptome Profile of the Chicken Thrombocyte: New Implications as an Advanced Immune Effector Cell

**DOI:** 10.1371/journal.pone.0214895

**Published:** 2019-04-01

**Authors:** Farzana Ferdous, Christopher Saski, William Bridges, Matthew Burns, Heather Dunn, Kathryn Elliott, Thomas R. Scott

The BioProject accession number in the Data Availability statement is incorrect. The correct accession number is: PRJNA344071.

## References

[1] Ferdous F, Saski C, Bridges W, Burns M, Dunn H, Elliott K, et al (2016) Transcriptome Profile of the Chicken Thrombocyte: New Implications as an Advanced Immune Effector Cell. PLoS ONE 11(10): e0163890 10.1371/journal.pone.0163890 27711235 PMC5053482

