# Supplementary figures and images for: Transcriptome Profile of the Chicken Thrombocyte: New Implications as an Advanced Immune Effector Cell
Source: PLoS One. 2016 Oct 6;11(10):e0163890. doi: 10.1371/journal.pone.0163890 (PMC5053482; doi:10.1371/journal.pone.0163890)

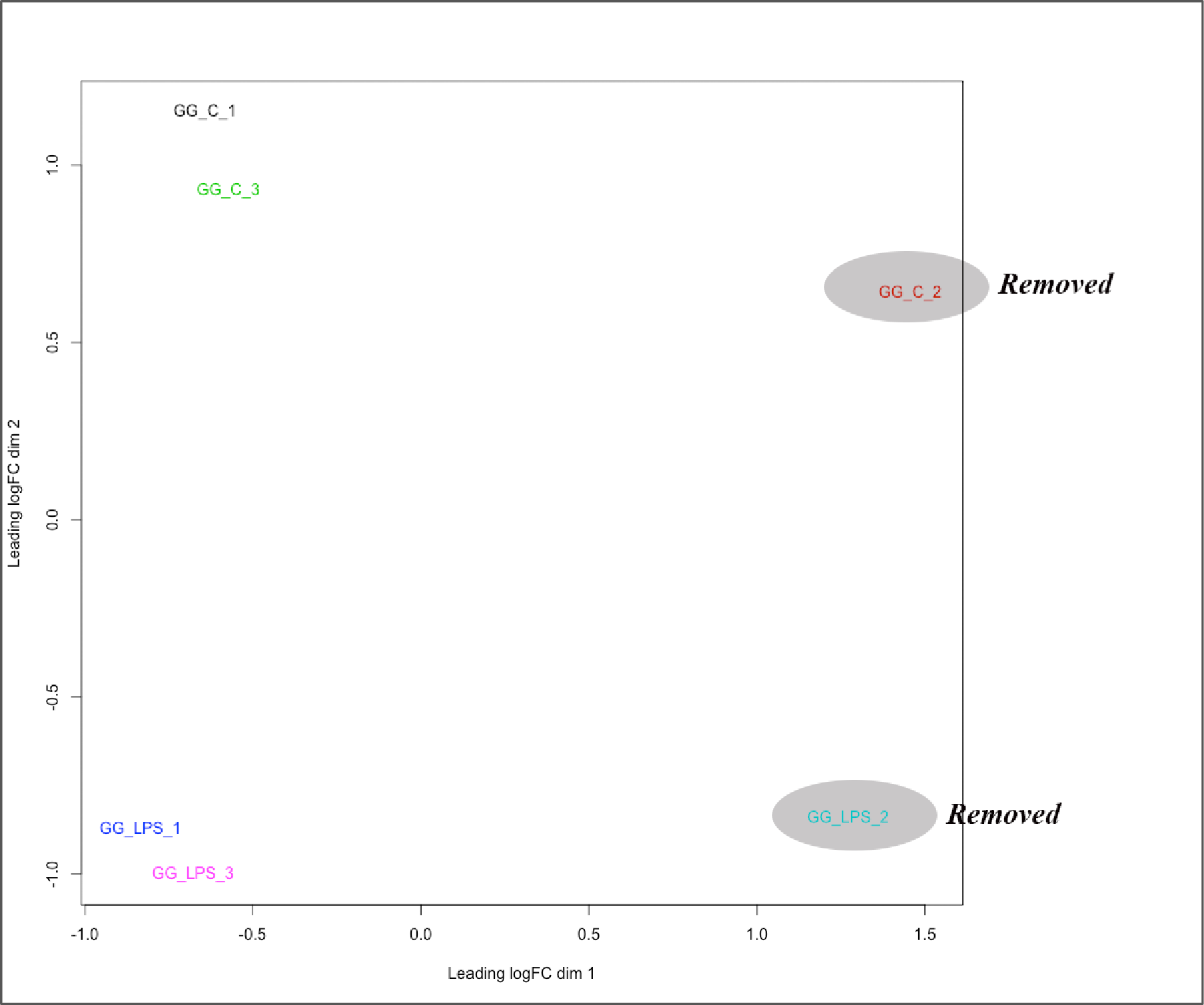

Supplement: S1 Fig — (TIF) [file pone.0163890.s001.tif]
